# Building resilience by challenging social norms: integrating a transformative approach within the BRACED consortia

**DOI:** 10.1111/disa.12341

**Published:** 2019-04-04

**Authors:** Chesney McOmber, Camilla Audia, Frances Crowley

**Affiliations:** ^1^ Post Doctoral Associate University of Florida United States; ^2^ Research Fellow, Geography, School of Global Studies University of Sussex United Kingdom; ^3^ Learning Coordinator and Research Assistant, King's College London United Kingdom

**Keywords:** BRACED (Building Resilience and Adaptation to Climate Extremes and Disasters), climate change, gender, gender transformative approach, resilience, transformation

## Abstract

Resilience is a complex phenomenon whereby a multitude of social and environmental factors, including gender, combine to shape the ways that shocks affect people. Looking at two BRACED (Building Resilience and Adaptation to Climate Extremes and Disasters) projects, in Burkina Faso and in Ethiopia, this article uses a desk review and primary data from partners and people at risk to explore how a gender‐transformative approach can be an integral part of resilience‐building projects, particularly those implemented by multi‐stakeholder consortia. It also suggests ways to incorporate a stronger gender component in similar future projects. The article argues that donors and programme managers must provide clear principles and guidelines for achieving gender equity within resilience‐building efforts. However, these must allow flexibility to adapt to norms, needs and resources as determined by implementing partners. The right balance can be achieved by facilitating spaces for individual and collective goal‐setting; assessing current capacity and trajectories; and lesson‐sharing as an iterative process for institutional learning.

## Introduction

Learning the best methods to build resilient societies is critical to facing the global climate challenges of our times. Doing this well requires a multi‐faceted approach to development programming, linking vulnerability, social equity and a transformation of the systems and structures that enforce this vulnerability (Walker et al., 2004). One point of entry to strengthen resilience has been gender. Increasingly, development institutions seek to improve empowerment through gender‐transformative approaches, which aim to challenge the patriarchal structures and norms that shape and reify conditions of vulnerability for women (Smyth and Sweetman, 2015). Pelling (2010) questions whether adaptation efforts are actually aimed at ‘accommodating risk and its root causes rather than at the root causes themselves'; Béné et al. (2012) crucially emphasise the inclusion of a pathway or approach that seeks to challenge and transform the status quo. This requires research that enables us to understand how to deliberately transform systems and social structures (O'Brien, 2012).

The debate is still open on how best to bring about such transformative change for resilience‐building, including how best to grasp the complexity of resilience where livelihoods, social norms, gender differences and vulnerabilities are inextricably tangled and still not well understood (Duckett et al., 2016). It is imperative, then, to understand if and what types of organisational mechanisms are helpful in supporting the type of transformative change that builds resilience, and those that undermine this process. The BRACED (Building Resilience and Adaptation to Climate Extremes and Disasters) work on resilience provides an avenue by means of which to explore such mechanisms within consortia.

This article explores the efforts the BRACED programme has made to address gender equity as a mechanism for building resilience within its project activities. Drawing on two projects within the BRACED programme, Zaman Lebidi (meaning “The world is changing”) in Burkina Faso and Climate Information and Assets for Resilience (CIARE) in Ethiopia, it explores the ways in which organisations, particularly those that operate in a complex consortium of partners, work to integrate a gender‐transformative approach towards building resilience. BRACED began its work with a broadly defined gender intervention that sought to address gender inequalities with the aim of mitigating the social and structural factors disempowering women.

This initial language was vague, perhaps intentionally, so as not to limit the types of gender interventions that could be implemented. This built‐in ‘flexibility', however, left the BRACED implementing partners with little common direction on how to challenge gender norms, or without the tools to do so effectively. This led to differences across partners following internal gender‐related procedures.

As research and learning partner of the Zaman Lebidi and CIARE projects consortia, King's College London (KCL) examined how gender affected people's resilience. As part of this effort, KCL facilitated spaces (workshops, fieldwork exercises) to bridge the gaps in knowledge and planning across implementing BRACED partners in order to respond to the gendered needs within communities. A review of the evolution of the two BRACED projects identifies some shortcomings that hindered initial efforts to coordinate a gender‐transformative approach to building resilience across a large consortium. We argue that a lack of time, resources and spaces for discussing a common approach to gender as a pathway to build resilience presented challenges, delays and incoherence across the consortia about how gender issues were addressed over the course of the projects. This led to the implementation of gender‐sensitive approaches rather than the initiation of more transformative change, which, we argue, is a fundamental pathway to resilience.

The first part of this article addresses the questions: What is resilience? How does gender transformation relate to resilience? And why is gender equity an effective avenue by means of which to pursue transformation? The second part explores the BRACED gender approach and how it evolved over the course of the projects. These observations also apply to initial challenges that emerged in trying to integrate transformative approaches into the gender mainstreaming agenda for BRACED project activities.

We ultimately conclude that gender‐transformative approaches implemented within a multi‐stakeholder consortium such as BRACED are better achieved when the following three conditions are met. First, it is imperative that underlying guidelines and principles for gender equity are clearly articulated within the broader mission of the ‘big picture’ project goals. All partners within the consortia should understand clearly why gender equity is imperative and integral to resilience‐building outcomes, what is meant by transformation and what success ultimately looks like—that is, what gender equity outcomes are expected at the end of the project.

Second, these guidelines and principles should allow for flexibility in order to be able to accommodate and respond to the contextually defined norms, needs and resources for each implementing partner. Gender inequalities are experienced differently across varying sectors of society, even within a single village. Implementing partner institutions are operating within different structures of power, and with varying levels of resources and capacity and different ideologies, which means that outcome goals—and the pathways to achieving these—may be very different over the course of a project.

Finally, finding a balance that allows for both an effective programming structure and flexibility to adapt to the local context in order to promote transformation requires an iterative process of feedback loops, self‐reflection and assessment and institutional learning within and among the consortia partners. Where opportunities for such collaboration and learning on the implementation of gender programming can exist recurrently throughout the course of the project—from its inception to its completion—institutional learning can flourish. This provides opportunities to continuously strengthen efforts to integrate transformation into gender mainstreaming initiatives and, ultimately, improves the chances that development interventions will advance gender equity in sustainable ways, building the resilience capacity of people at risk.

## Project organisation and study justification

### BRACED programme context and the CIARE and Zaman Lebidi projects

Running from early 2015 until December 2017, BRACED was established by the UK Department for International Development (DFID) to help people become more resilient to climate extremes and disasters in South and South East Asia and the Sahel region. It formed a network of 15 projects across 13 countries. The programme also benefited from a consortium of Knowledge Manager (KM) organisations led by the Overseas Development Institute (ODI).

KCL acted as the academic partner of two BRACED projects: Zaman Lebidi in Burkina Faso; and CIARE in Ethiopia. Zaman Lebidi aimed to build the resilience of 1.3 million people in 4 provinces in Burkina Faso with a consortium of 6 partners (see Box 1). The CIARE project sought to increase the resilience capacity of 700,000 people in 12 *woredas* (districts) in Ethiopia with a consortium of 10 partners (see Box 2). Both consortia were led by the international aid agency, Christian Aid, which was tasked with the role of project management unit. Both aimed to support local agro‐pastoral communities to overcome the negative impact of climate change through improved access to and usage of climate information services and integrating this with resilience‐building activities such as agricultural training and livelihood diversification projects. The projects brought together stakeholders who operate at different levels (local, national and international) and in different areas of expertise: international and local non‐governmental organisations (NGOs), meteorological agencies and other local authorities, communications specialists and research institutions. With a mandate for research and learning, KCL's role was to contribute to filling a knowledge gap on best practices around building resilience.

**Box 1. Project actors and activities in Burkina Faso**

**Christian Aid (CA)**: main implementing partner with oversight for the work of the programme

**Oxfam Intermon (IO)**: implementation of project activities in the Centre North region

**Action Contre la Faim (ACF)**: implementation of project activities in Gnagna province, East region

**Alliance Technique d'Assistance au Développement (ATAD)**: design and delivery of strategies and activities to cope with climate shocks

**Office de Développement des Eglises Evangéliques (ODE)**: technical assistance to communities and data collection in the field

**Internews Europe**: production and dissemination of climate information. Working in partnership with Burkina Faso national radio (RTB)

**King's College London (KCL)**: thematic research, production and dissemination of policy briefs and academic papers

**Met Office (UK Met)**: provision of weather and climate advice

**Burkina Meteo (Météo BF)**: local partner for the Met Office, providing weather and climate advice

**Radio Télévision Burkinabé (RTB)**: production of regular weather forecasts for local radio

**Source**: authors.

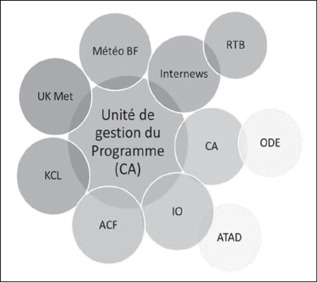

**Source**: authors, based on Rigg at al. (2015).

Intervention zones in Burkina Faso

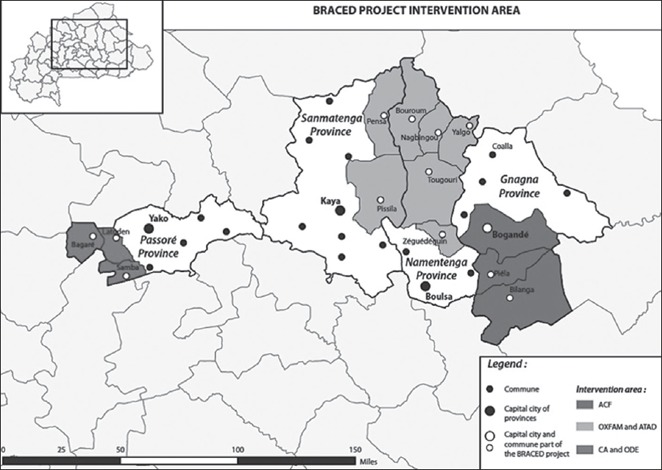

**Source**: authors, based on Rigg at al. (2015).

**Box 2. Project actors and activities in Ethiopia**

**Christian Aid**: main implementing partner with oversight for the work of the programme

**BBC Media Action**: design and implementation of the communications strategy, focused on local radio

**ActionAid Ethiopia**: implementation of practical resilience activities in Seru and Kombolcha

**Hundee**: implementation of project activities in Yabelo and Arero *woredas*

**SOS Sahel Ethiopia**: implementation of project activities in Oromia and Southern Nations, Nationalities and Peoples Region (SNNPR)

**Action for Development**: implementation of project activities in Oromia and SNNPR

**Women Support Association**: implementation of project activities in Konso *woreda*

**King's College London**: thematic research and monitoring and evaluation (M&E) of outputs

**National Meteorology Agency (NMA)**: development and implementation of systems and processes to improve climate forecasting and user‐friendly climate information for at‐risk populations

**Met Office (UK Met)**: support to NMA to develop systems and processes necessary to improve climate forecasting

**Source**: authors.

Intervention zones in Ethiopia

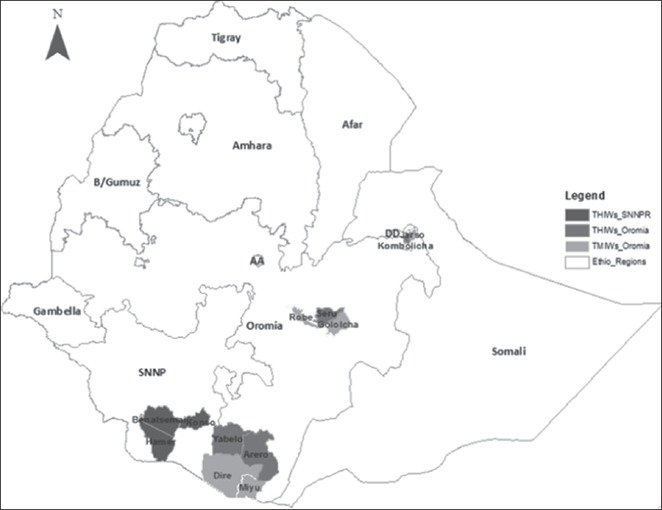

**Source**: authors, based on Rigg at al. (2015).

KCL's role was also to facilitate learning; the team achieved this by participating in regular Skype calls and meeting, discussing policy briefings and learning papers and organising learning events and workshops. Part of the research for this paper comes from interviews, focus group discussion, participant observations, feedback and conclusions of a workshop held in Ouagadougou in December 2016 for Zaman Lebidi project partners. The workshop was titled ‘*Towards a gender‐transformative approach in BRACED*', and aimed to establish a road map for a gender‐transformative approache while the programme was ongoing. Participants from all implementing partners (NGOs, researchers, communication agencies, Burkina Met Office) spent three days reviewing approaches, theories and tools to integrate gender in resilience‐building projects; carrying out focus group discussions in the villages to gather data supporting these theories; and mapping out an action plan for the last year of the project ahead. Findings from the workshop substantiate our arguments and are presented later in the article.

### Viewing transformation through the lens of resilience and systems thinking

In simple terms, resilience means the ability of a system (individual, household, village, population, etc.) to withstand and continue to function against shocks or stresses (Béné et al., 2012). It originates from the field of ecology, where it describes the quality of an ecological system to ‘bounce back', change, reorganise and restore its usual functions in the face of a threat or disturbance (Walker et al., 2004; Martin‐Breen and Anderies, 2011; Berkes, 2017). In the context of climate change and disasters, the Intergovernmental Panel on Climate Change (IPCC, 2014) defines resilience as follows:

*… the capacity of social, economic, and environmental systems to cope with a hazardous event or trend or disturbance, responding or reorganizing in ways that maintain their essential function, identity, and structure, while also maintaining the capacity for adaptation, learning, and transformation*.

Many narratives of resilience recognise that people at risk have a strong basis and resourcefulness to overcome adversity (Smyth and Sweetman, 2015), but it is even more crucial that this group uncover and challenge power relations to be able to address hazards while giving appropriate attention to the social context (Wisner et al., 2003).

Because the nuances of power and social relationships are highly contextual, resilience‐enhancing interventions must respond to a unique configuration of vulnerability specific to the needs of individual communities. This means the capacity to enhance people's resilience can vary greatly from one household to the next (McDaniels et al., 2008; Bahadur et al., 2015). Even within the same community, resilience capacities may be expressed differently according to gender, age, ethnicity, caste, social class or income and, subsequently, people will have developed different strategies to absorb and adapt to shocks. Costs and benefits associated with environmental issues are distributed unequally worldwide, linked with political, social and economic differences in power. Thus, it is important to consider the notion of power in human interactions, as some policies may bring advantages to some groups but not others (Pike et al., 2010).

Resilience thinking can fall into the trap of placing too much emphasis on the stability of a system as a whole rather than examining the structural and historical causes of vulnerability and power imbalances that arise at different scales. Indeed, Berkes (2008) argues that a focus on systemic growth in economic terms, rather than changes in social relations, science or new technology, distracts from the interesting ways in which innovation can shape the prospects for resilience in communities. This poses an ethical question for people involved in improving resilience: is this *status quo* something that should be maintained (Cannon and Müller‐Mahn, 2010)? It is in the context of this academic debate that BRACED has implemented development interventions to build resilience through transformation.

### Resilience to climate extremes and disasters in BRACED

In the BRACED programme, resilience is understood as the capacity to *adapt* to, *anticipate* and *absorb* climate extremes and disasters—the so‐called ‘3As’ approach (Bahadur et al., 2015). This approach, adopted early on in the project design, also acknowledges the growing discourse on transformation as defined by Béné et al. (2012). It recognises that goals of resilience are best achieved when responses to environmental crises are carefully tailored to the complexity of social relationships. This means that every development intervention needs to take into account social norms, carefully consider the social inequalities inherent to these norms and acknowledge and address complex and diverse needs of all of the people at risk whose resilience is being built and strengthened (Rigg et al., 2015).

In fact, communities can build more resilient systems capable of responding to climate change through sustainable transformation of social equity. This is sometimes referred to as ‘transformative capacity', which is the ‘[socio‐ecological system's] capacity to create a fundamentally new system when ecological, economic or social structures make the existing system untenable’ (Béné et al., 2012). This can be initiated by recognising the mechanisms that perpetuate inequality and by valuing and communicating knowledge from all stakeholder parties across the consortium. It requires challenging the root causes of vulnerability. Thus, resilience is about the ability not just to withstand or ‘absorb’ shocks but also to adapt to them if necessary, and, when this becomes no longer acceptable, to change the system completely. Resilience is a process that enables a system to absorb, adapt or transform in the face of shocks or stresses (Béné et al., 2012). Transformations can occur in multiple ways within systems, in science and technologies, institutions and power relations.

### Gender and rural resilience

Resilience within social systems requires careful attention to marginalised populations and a commitment to improving social inequalities. One critical area for opportunities to improve resilience is by addressing the structures of gender inequality that persist throughout the world. Particularly in rural areas, where women are major producers of world food (FAO, 2011), inequalities in women's access to resources and decision‐making roles hinder their ability to respond to environmental crises.

An International Food Policy Institute (IFPRI) report estimated that women in Sub‐Saharan Africa could account for more than three quarters of the agricultural labour force (Quisumbing et al., 1995). Still, this number is merely an estimation, because women's work is often unpaid, unrecognised and therefore under‐accounted for (Dankelman and Jansen, 2010; FAO, 2011). Women's agricultural work is often one element of a triple burden of labour (productive, reproductive and community managing) that usually differs greatly from men's roles and responsibilities in the house and community (Moser, 1993; FAO, 2011). Yet it is precisely because women are doing much of the agricultural work—albeit often not reaping the economic benefits—and because they are often invisible members of the agricultural workforce that attention to their vulnerability is essential to building and strengthening resilience among vulnerable populations. Gendered power hierarchies (and the structures and norms that emerge from these) have created a legacy of inequity that underlies and intensifies the impacts of severe climate events in ways that are ‘differentiated’ and ‘distinct’ (Carr and Thompson, 2014). Women's global experiences of inequity and oppression—especially for those whose livelihoods rely heavily on subsistence agriculture—have made them particularly vulnerable to the impacts of environmental crises (Denton, 2002; Goh, 2012; Carr and Thompson, 2014).

Despite their important contribution to household livelihood strategies, women in developing countries are often excluded from efforts to improve resilience to climate shocks and disasters. On a global scale, men are more likely to be landowners than women (Agarwal, 2018; Deere and Leon, 2003). As such, men are often considered the primary producers and decision‐makers within their households. And, while this is sometimes the case, the fact is that gendered roles regarding decision‐making and division of labour are much more nuanced. Still, these assumptions have led to the mistaken expectation that women are not also fulfilling these roles. This has resulted in a weaker understanding of women's agricultural needs—and a need for recognition that those needs may be different from those of their male counterparts (Perez et al., 2015). In reality, women's vulnerabilities are different from men's because their roles and responsibilities within the household and community are different (Dankelman and Jansen, 2010). Bob and Babugura (2014) argue that the exclusion of women in activities to adapt to or mitigate climate risks means there is untapped potential in development efforts. In many countries, women are heavily involved in climate‐sensitive activities (farming, forestry or fisheries for home consumption or for sale) and consequently have accumulated critical, but often undervalued, knowledge about environmental protection adaptation (Dankelman and Jansen, 2010).

In a literature review on gender‐differentiated impacts of climate change in developing countries, Goh (2012) finds that climate‐related events affect men and women's well‐being and assets differently. Overall, climate‐related shocks appear to affect women more negatively than they do men. In general, this is because of social and cultural norms regarding gender roles and women's lack of access to and control of assets (ibid.). These impacts are often intensified through limited access to particular types of capital—natural, human, social, physical, financial and political—making women dependent on male household members to sustain and make changes to their livelihoods and less prepared to respond to severe weather events and climate change (Meinzen‐Dick et al., 2014). In households where gender shapes the allocation of resources and roles of responsibility, women may be unable to negotiate on an equal footing with their male counterparts. This means that, even if women have equitable access to information, they are unable to translate that information into actionable change. This is particularly relevant to the BRACED project, where households received training and access to scientifically generated climate and weather information.

This research explores how lack of understanding of the reasons for implementing gender‐transformative approaches, combined with a lack of guidance on how to integrate gender within the broader framework of building resilience, led to limitations in how BRACED projects tackled gender inequities as a holistic component of improving resilience.

### Building resilience through gender‐transformative approaches

Women have the potential to be critical contributors to improving food security, livelihoods and resilience because of their heavy involvement in agriculture and in assuring household well‐being (Quisumbing et al., 1995). However, since social inequities continue to inhibit women's full ability to build resilient livelihoods, development institutions have worked to integrate gender approaches into their programming to address these. Gender mainstreaming refers to the process of incorporating the knowledge, experiences and interests of both women and men into a project, to ensure both can influence, participate in and benefit equally (International Labour Organization definition, in Ginige et al., 2009). While the integration of gender mainstreaming practices within institutions has been beneficial in some ways (such as providing opportunities for gender trainings and the development and diffusion of gender tools for analysis), others feel that this approach has resulted in disappointed hopes for improved gender work within development (Cornwall et al., 2007). Critics argue that gender mainstreaming has resulted in gender inequality treatment that precludes the critical and structural change necessary to challenge the systems of patriarchy that underlie them. Kabeer (2005, p. 16) writes, ‘We are, therefore, interested in transformative forms of agency that do not simply address immediate inequalities but are used to initiate longer‐term processes of change in the structures of patriarchy'. Gender mainstreaming, instead, should have transformative ambitions.

Gender‐transformative approaches ‘seek to foster change in individual capacities (knowledge and skills), attitudes, agency and actions; the expectations embedded within relationships between people in the home, in groups and in organisations; and institutional rules and practices’ (Cole et al., 2014, p. 8). Development institutions are increasingly employing these approaches as they work to implement more gender‐equitable programming (Jost et al., 2016). This is a long‐term and ambitious endeavour, and one that exceeds the scope and timeline of BRACED. Still, aligning project activities with this approach is an important first step in pursuing sustainable gender equity in the participating communities (Christian Aid, 2014b, 2014a). Centrally important to the achievement of gender‐transformative approaches is attention to the context shaping the gendered power hierarchies; this is equally important to resilience‐building and a crucial point of the BRACED projects. There is not one model to resolve gender inequity but many, depending on the norms of each particular society (Kantor et al., 2015). It is also critical that these processes include both women *and* men. Drawing on the epistemological approaches embedded within gender‐transformative approaches, achieving true gender equity relies on the recognition that building resilient systems extends beyond the mere provision of gender‐equitable climate information, or integration of women into economic activities. Instead, it is all these things together; it is the interworking of the economic, the social and the psychological that must be addressed in their context.

## Methodology

This article focuses on exploring how gender approaches were considered, integrated and implemented over the course of the two BRACED projects described above. To gain an understanding of how external development agencies may influence transformation, KCL carried out interviews with consortia member staff based at project sites and in country office headquarters, either in person or via Skype, between October 2015 and June 2017. The aim of the interviews was to understand partners’ perceptions of programme approaches and household‐level findings related to gender.

Ten partners were interviewed twice in Burkina Faso, with a target of at least one person per implementing organisation and programme management unit. Given the projects’ high turnover, only four of the partners in Burkina Faso were interviewed in the initial stages of the project. Fourteen people were interviewed in Ethiopia and seven people based in the UK. At the village level, semi‐structured interviews and focus group discussions were carried out with 32 households in the Ethiopian *woredas* of Seru and Kombolcha, and 24 households across the Burkinabe provinces of Passoré, Sanmatenga, Namentenga and Gnagna. Methods also included participant observation in villages and households and qualitative in‐depth interviews at household level.

Findings are also drawn from six interactive workshops facilitated by KCL over the course of the BRACED projects, in Burkina Faso, Ethiopia and London. Data also includes notes and participant observation from fieldwork exercises that took place as part of a gender‐focused workshop. The research findings have been triangulated with observations dating from the start of the project development phase in 2014 and with the analysis of key policy documents, including the project logical frameworks, M&E plans and the project's theories of change.

The researchers here recognise biases that these methods of data collections carry with them. The main biases of this study include the small sample of in‐depth interviews, which makes it difficult to generalise conclusions to other socio‐cultural contexts, and the limited timeframe, which makes change difficult to observe over the data collection period. These have been partially addressed through the analysis of project documents, especially initial proposals and final evaluation reports.

In addition, while approaching colleagues was advantageous in that there was already a basis of trust and a common understanding of the project and organisational dynamics, it also meant the team was well‐known to the interviewees, which could influence the degree to which information is shared. To counteract this bias, all participants were carefully informed that their information would be used for publications and analysis of the BRACED projects dynamics. There is also a bias when collecting data at the village level when researchers are perceived as partners of a specific project or organisation; interviewees may feel that the information they share could affect their relationship with associated development actors. To partially address this bias, researchers clearly outlined the objectives of the research, participants’ anonymity and the non‐operational role of KCL within BRACED.

The following sections detail the efforts the BRACED programme has taken to address gender equity as a mechanism for building resilience within its projects’ activities and the lessons learned from and across the BRACED Zaman Lebidi and CIARE consortia.

## Findings and discussion

### Setting clear intentions for a gendered approach to resilience‐building

Ambitions for gender transformation were not clearly defined at the onset of the projects and this led to challenges in the overall ability to achieve such ambitions within the short timeframe of the projects. Initial application documents for both the Ethiopia and Burkina Faso BRACED projects stated that:

*Identifying and analysing internal and external dynamics that lead to changing vulnerabilities, risks and opportunities should be incorporated into programme design and activities regularly reviewed allowing for flexibility in programming. This enables equity, the first principle of resilience, as understanding these changes are key to assuring that the programme engages with the most vulnerable at any given time* (Christian Aid, 2018).

Furthermore, it was stated that project activities would identify gendered roles and responsibilities within stakeholder communities and understand the ways in which these affect access to and utilisation of resources, with particular interest in equitable access to climate information (Christian Aid, 2014a, 2014b). From the beginning, gender is not specifically mentioned in the theory of change for either partner country. While this means there is potentially a great deal of opportunity for adaptive innovation in integrating gender into programming and transforming gender norms, it also bears the risk that gender approaches will be neglected or inadequately integrated.

The proposal puts forwards that the project will ‘address the inequalities identified and meet the specific needs of women’ (Christian Aid, 2014a). Embedded in this statement, then, is an allusion to the need to transform those obstacles that lead to gender inequalities in order to improve women's access to financial, human, social, political and material resources. One document, written early in the project timeframe, explains that a gender approach should prioritise ‘that gender sensitive programming and that women and girls are reached; that gender specific resilience building plans are identified and implemented; that women are involved at all stages of the programme cycle and in key decision making’ (Christian Aid, 2014a). Arguing for a more democratic and participatory process in the progression of project activities and outputs—as it pertains to gender equity—another early planning document prioritises ‘horizontal dialogues’ as opposed to ‘vertical dialogues’ within the institutions as a way of mitigating some of the *status quo* power hierarchies perpetuating gender inequity (Christian Aid, 2014b). Still, what this should entail in practice remains vague.

While the proposals point to the need for a gender approach, the projects lacked any specific gender policy document presenting a fundamental road map for achieving this. Furthermore, M&E processes did not prioritise or reinforce these initial desires for nuance in project benchmarks and outputs. Mid‐project reports present very little data on gender and barely any analysis on what that data means for women's experience of gender equity and opportunities for resilience. Ultimately, the projects lacked a ‘gender roadmap’ from the very beginning, and this is evidenced by the lack of a gender contribution in the mid‐point reviews and later project documents.

This challenge was identified during the first year of the programme, and ODI, as leader of the KM consortium, led a multi‐country study to reflect on challenges and successes of early implementation of gender‐related activities in the BRACED programme (Le Masson, 2016). This initial working paper on early BRACED activities highlighted that the intention was that the programme would support women and girl's empowerment. However, it remained unclear on the approaches to be taken to achieve this goal. At the same time, it revealed gaps in institutional roadmaps and leadership in terms of how best to navigate the tension between the obligations of short‐term outputs and analysing long‐term social transformations.

The KCL team built subsequent research from the findings of this initial gender review and working paper. KCL framed semi‐structured interviews for partner institutions in response to the initial findings, focusing on Burkina Faso and Ethiopia's Christian Aid‐led projects, in order to ascertain a more complete understanding of the processes for strengthening resilience through gender as practised within BRACED.

With little direction as to expectations of what gender equity should look like at the end of the project, what types of timelines such interventions should follow and what methods should be employed to attain these goals, we found that activities were unable to live up to the initial expectations of the proposal, or to fully grasp what those expectations truly were to begin with. The challenges that emerged, from very early on, with regard to gender programming within BRACED centred on this initial challenge: that a gender‐transformative approach, while implied, was not clearly articulated or integrated throughout the consortia. The following excerpts from interviews with partner institutions highlight some of this disjuncture.

*You need to be guided by the overarching aim of the global BRACED programme which needs to specifically address women and girls. Unless you have a separate measurement framework you cannot prove you have specifically addressed the issues* (BRACED Ethiopia partner, 2016; Skype interview).

*I think the main aim objective of the overall project is to make sure we increase resilience and we make sure women are a key part of the population we want to reach but I don't think we are addressing gender issues, we target women in everything we do but we don't challenge any social … whatever* (BRACED partner, 2016; Skype interview).

*Yes, that is true that it [gender] does not feature in the theory of change … it is an ‘oubli’ an oversight—it should not have been like that—it is mainly a fault in the diagram as it is integrated into the log frame. But it is significant that it is not there as we should have noticed* (BRACED partner, 2015; Skype interview).

### Conducting gender analysis

BRACED developed quantitative and qualitative tools to monitor different aspects of resilience‐building and evaluate the impact of the project. These tools included initial vulnerability assessments, M&E baselines, inception reports and qualitative research, including that led by KCL in the Zaman Lebidi and CIARE consortia. They all looked into gender equity from different angles and with different aims and goals. The BRACED Participatory Approach (BRAPA) was an initial benchmark assessment of livelihoods, vulnerabilities and resources regarding climate extremes and shocks. While some of the questions were gender‐disaggregated and helped to reveal a quantitative assessment of gendered access to information, the tool did not allow an understanding of the underlying mechanisms that prevented women having equal access to and utilisation of climate information and subsequent decision‐making dynamics. BRAPAs did have qualitative tools such as focus group discussions and open‐ended interviews; however, their results and analysis could not be properly inputted into the projects’ structure, owing to tight deadlines; however, further analysis of some of the data proved useful for some of the KCL‐led research.

Initial project applications for both Burkina Faso and Ethiopia speak to some of the complexities of measuring gender equity and transformation. And yet the metrics applied through quantitative ‘gender‐disaggregated’ data, like the BRAPA, are not capable of revealing such nuances. The documents explain:

*It is therefore important to disaggregate to the household and individual level throughout the baseline assessments and PVCA [Participatory Vulnerability and Capacity Analysis] process in order to capture the complexity of different communities, understand the specific challenges and opportunities relating to women and girls and other marginalised social groups and understand relationships between individual, household and community level interactions. It is also important to incorporate the understanding of a community as: a heterogeneous entity with porous boundaries with a range of complex and fluid internal and external dynamics and multi‐scalar relations at play in order to ensure equity, accountability and contextualised resilience‐building activities* (Christian Aid, 2014a).

The BRAPAs, however, presented an incomplete understanding of gender power relations within project sites. Among the challenges inherent in this type of assessment is that they may lead to policies that satisfy project objectives of integrating women more fully into a project but do not address the nuances of power relationships under the types of circumstantial pressures so critical to developing resilience. For example, a man may make decisions regarding crop planting, except during a particular time of the year when he migrates for work and the wife makes the decision in his stead. These exceptions are considered not centrally important to overall patterns of gendered participation and decision‐making. Yet, in moments of hardship, households adjust to meet livelihood needs—and in these moments roles and responsibilities can also adjust—particularly as they pertain to gender. Recognising that this happens, *how* it happens and what resources are necessary to support communities in these moments of hardship is critical to building resilience and the aims of the BRACED project. Carrying out BRAPA‐type assessment over a longer period of time, asking questions at different times of year and having time and resources to carry out full analysis and follow‐up of the data before inputting it into project design are key to ensuring projects target needs identified by people at risk. Moreover, built‐in flexibility in assessments and M&E tools could help unveil mechanisms or power relations that are key to identify entry points for transformation and resilience‐building.

Different tools were needed to uncover the nuances of cultural norms and the historical legacies of gendered roles and responsibilities. To partially address this gap in knowledge, KCL designed semi‐structured interviews, carried out with male and female members of selected households in BRACED‐targeted villages in Ethiopia and Burkina Faso. Qualitative assessments highlighted that men and women stated that decisions regarding sowing and harvesting (type of crops, timing, soil and water activities, field rotation, etc.) were usually discussed as a household; however, men always seemed to have the last word. Interviews also revealed that men were more likely to have access to a radio as well as to spend more time in the market place, and therefore greater access to scientific climate and weather information, including through informal discussions on rains and agriculture with people from neighbouring markets and villages.

One of BRACED's resilience‐building activities consisted in providing women's groups with goats; this challenged gender norms, as women normally do not own goats in the affected communities. The consequences were that women took on decision‐making roles and responsibilities with regard to this new livestock asset—roles and responsibilities that are traditionally reserved for men. While partners viewed this as a transformation in social norms, more in‐depth conversations during the focus groups revealed that this activity had not led to a clear outcome or change in decision‐making power.

*Elle dit qu'elle peut aller au marché vendre ses chèvres si elle veut mais elle demande la permission á son mari par respect et parce que c'est comme ça. Si elle gagne 10, elle remet 5 á son mari aussi. Mais elle ajoute qu'elle peut faire ce qu'elle veut avec l'argent qui lui reste*.

[She says she can go to the market sell her goats if she wants to, but she will always ask her husband's permission out of respect and because that is how you do it. If she gets 10, she will give her husband 5. But she added that she could do what she wants with the remaining money] (Woman, Tallé Mossi, Burkina Faso, December 2016).

*We give our advice, but we have limited involvement because we are engaged in so many household activities* (Woman, Lemiti Dindin, Ethiopia, November 2015).

*On écoute tout le monde parce que c'est comme ça, le monde a changé et les femmes ont le droit de parole. On essaye de prendre les meilleures décisions pour le ménage et pour les champs. Mais au final, c'est mon mari qui prend la décision, ça a toujours été comme ça*.

[Everybody listens to everybody to because that is the way it is now; the world has changed, and women have a right to speak up. We try to make the best decisions for the household and for the fields. But, in the end, it's my husband who will make the decisions, it's always been this way] (Woman, Pella, Burkina Faso, April 2017).

Across these examples, we see there are spaces for women's involvement in decision‐making processes. However, men are usually in at least partial control of this decision‐making power and the resources involved in these activities.

It is important to understand the complexity of these decision‐making processes among household members. The nuance is complicated: the man has a role in deciding what is to be done with household resources, but women also have a role to play in that decision‐making. Improving women's access to information may help improve their ability to negotiate a more influential role in household decisions. At the same time, women explained that they did make decisions about some of the household income, and, again, improved access to information may help them make informed investments of that money. This raises the following questions: What information needs to be made available to women in response to their specific needs in their specific communities? And how do those information needs differ from those of men in the community? Effective programming for transformation requires accurate identification of the complexity of power relationships within the household and community. It then requires careful assessment of what must be changed in order to improve equity. Finally, it requires careful consideration of what changes will lead to transformation that improves resilience.

### Establishing a common gender approach

Interviews at the partner level revealed that there was a real need for a common approach to resolving gender inequities. Initial KCL assessments identified that the gender interventions were more about gender analysis and less about transformation. So, while the initial intentions of the project anticipated pathways to more transformative change with regard to gender power relationships, in practice, partners had not explicitly integrated such goals into implementations. Most partner NGOs have gender goals within their usual modus operandi; this mostly means they follow internal guidelines rather than a commonly agreed approach. While attempts were made to collaborate and arrive at a common approach between partners (gender workshops were held in Burkina Faso among partners), a combination of the short timeframe of the project, lack of resources and time spent creating common understanding of procedures and harmonising approaches led to a disconnect between what was in the proposals, theories of change and log frames and what could effectively be implemented.

Where collaboration and exchanges did happen, these seemed to take place in informal or unstructured contexts. One such example is in Ethiopia, where partners with expertise and experience of women's self‐help groups and savings and credit associations were able to impart expertise and practical support through informal discussion and carrying out activities with other partners at the community level (Crowley et al., 2018). This was seen to significantly improve implementation practices and, ultimately, result in more impactful ways of strengthening resilience among people at risk. In Burkina Faso, one of the more fruitful spaces for collaboration was surprisingly observed to occur on a bus ride after a gender workshop field excursion. It was here that partners shared and discussed what they had learned from that day's field research. These discussions built upon the previous day's learning regarding gender approaches and how best to achieve transformation, applying what they had learned from the community focus group discussions that day. The important piece here was that the informal setting allowed partner institutions to engage in lively conversation *with each other* about gender. While conversations were happening within institutions about gender approaches, this was really the first time that all project partners were able to facilitate a discussion about gender strategy across the consortium after being presented with clear definitions and having developed a common understanding.

These conversations led to the final goal of the gender workshop (*'Towards a gender‐transformative approach in BRACED'*), establishing a road map for a gender‐transformative approach. The workshop's third and final day provided space for partners to come together to set collective goals and visions in this regard. This collaborative process was critical to achieving a collective transformative approach. Participants developed an action plan, with different steps to take to ensure that strengthening the resilience of people at risk from climate extremes included specific attention to gender and social norms. Partners implemented some of these actions, such as reinforcing Early Warning Committees in communicating and explaining climate information, and radio distributions across villages, specifically targeting women, to ensure means of accessing forecasts and agro‐meteorological advice. Others were more difficult to implement, such as using theatre to sensitise people at risk around gender inequities, since funds had already been committed to other activities. This illustrates an unresolved tension between resilience thinking that argues for transformation by challenging social norms by focusing on adaptive governance, learning and co‐production of knowledge, and what projects can achieve considering limited flexibility, complex consortia set‐ups and restrictions with regard to the allocation of time and resources.

More generally, the data analysis points to a lack of time and resources planned for and invested in creating spaces for interacting and learning from each other across the programme. In part, this owed to a lack of flexibility in project outputs and deadlines, which were already determined at the outset. On paper, the applications recognised the need for space to be flexible and to adapt to local needs and emerging vulnerabilities. However, the timeframe, difficulties in coordinating multiple and geographically distant partners and heavy bureaucratical and hierarchical structures meant this was difficult in practice.

Moreover, the M&E and learning tools enabled assessment of specific deliverables and indicators identified at the beginning of the project. While these tools were partially informed by the BRAPA data and initial inception reports, the lack of flexibility and precise focus made them inadequate to track tangential, unintended or unexpected changes or transformations outside the project's scope. We argue here that spaces, time and resources for flexibility are particularly central to adopting relevant, unified and efficient gender‐transformative approaches. Partners from both projects highlighted the need for more flexibility in assessing goals and reorganising priorities to align them with the timeframe and with the needs of those whose resilience they aimed to strengthen. In fact, bringing together different actors to discuss viewpoints, interpretations and tools to implement programme components, including gender transformation, is vital to create the basis for an ongoing iterative process of mutual respect and understanding, leading to the more holistic approach that is needed to build and strengthen resilience.

The lack of common approach was compounded by a gap in gender capacity within partner institutions. Turnover of gender expertise was high within partner institutions. Many who had received gender training had received it as many as five years prior, and as part of their organ isation's gender vision rather than targeted to a BRACED‐specific set of resilience‐building activities. This helped inform and promote the aforementioned workshop on gender‐transformative approaches in December 2016; however, the lack of feedback loops and flexibility led to difficulties in implementing workshop findings and recommendations within the project, as it was already being implemented and monitored.

### Recommendations

The review of gender throughout the two projects suggests that, for transformations to occur at individual, household and village levels, all members of the consortia must be involved and invested in learning, sharing and combining their knowledge. The broader research carried out by KCL points toward adopting and integrating methods of co‐production of knowledge early on within projects, in order to allow for flexibility and sensible allocation of time and resources throughout, acknowledging that, in a multi‐stakeholder setting such as a consortium, producing new knowledge needs to focus on valuing all sources and types of knowledge equally, aligning different priorities and finding spaces where working together at the same time is productive while recognising times to work separately. Clearly defined methods, responsibilities, ethics and principles for co‐production of knowledge are essential to disrupt current views of societies, social norms and, more generally, development interventions while allowing for all actors, first and foremost people at risk, to come together and create new knowledge useful to address relevant societal issues (Visman et al., 2018). It is important to point out that successful co‐production of knowledge recognises that those whose resilience we aim to build are an integral part of the consortium, alongside NGOs or academics.

Integration of trainings on methodological approaches that allow for qualitative evaluation of nuanced community needs is especially important. Methods of gender analysis are also essential to integrating transformative approaches in BRACED programming initiatives. Workshops with all partners should aim to discuss how and why social inequity occurs, encouraging local populations to identify social problems and involve people at risk as much as possible in the process of coming up with solutions to address them. Providing space for institutional learning across the BRACED consortia about best practices regarding information‐sharing and gender equity could also benefit projects’ attempts to promote sustainable change in resilience strategies for people at risk from climate extremes and disasters.

Finally, M&E criteria and processes are always bounded to the aims of the project, and many times this means that gender is recognised as a quantitative function (such as male vs. female participation in a given training, etc.) rather than as a system of relationships. This means that gender can be accounted for in the ratio of targeted men and women, for example, but will not be captured systematically as an element of transformation. Systematic assessment of transformative processes owing to gender is difficult to capture in such M&E measures because co‐benefits often accrue long after the project has ended and are not collected or observed.

Ultimately, this is the challenge of conducting development projects with transformational ambitions. Reconstruction of long‐held discriminatory structures and norms is often an intergenerational process that requires decades, not years, to reveal noticeable and meaningful transformations. The observable impact on resilience may take even longer. So, how can we know, over a course of a project, whether transformation is occurring? One way is to ensure that criteria for M&E processes can reflect this type of change qualitatively, to capture the complexity and nuances inherent in transformational processes. For example, within BRACED, mid‐term reviews revealed quantitatively the extent to which project activities had included women. This quantitative data on participation shows that target goals established at the outset of the project were met but cannot capture or illuminate the way in which women were engaged, and the specific ways in which their involvement affected their roles in the community and in the household (such as productive and reproductive decision‐making, time allocation, social capital, etc.).

Kabeer (2005) critiques M&E processes that focus on quantitative measures to resolve indicators of gender inequality. She emphasises the reality that empowerment may be occurring in ways that are unrelated or tangentially related to gender equity—and that this relationship may have different implications in different contexts. While women's earned income may increase their negotiating power within the household in some contexts, in other cases it may not. Quantitative indicators measuring women's participation in economic activity will therefore reflect gender parity in economic activities related to the project, but will not indicate whether this has been a benefit or a hinderance to women's overall empowerment. This is a challenge that many development projects have faced, as Kabeer observes in her critique of the Millennium Development Goals’ M&E measurements. Her argument seems to echo relevance to the BRACED project as well:

*Gender relations, like all social relations, are multi‐stranded: they embody ideas, values, and identities; they allocate labour between different tasks, activities and domains; they determine the distribution of resources and they assign authority, agency, and decision‐making power. This means that gender inequalities are multi‐dimensional and cannot be reduced to some single and universally agreed set of priorities. Any attempt to do so will run the danger of being either too narrow … or a wish list that is too long and complex to act on* (Kabeer, 2005, p. 23).

As development programming increasingly seeks to effect transformative change as a means of building resilience, the M&E process must also undergo its own transformational change. Indicators measuring deliverables and outputs must allow for transformative processes to be revealed and observed. Transformation is complex and nuanced and, as such, ensuring that there are measures within M&E processes, such as in‐depth qualitative interviews triangulated with more anthropological methods, to capture mechanisms of gender transformation is imperative to building resilience. Such methods and measures must account for micro‐level dynamics that are often expressed through slight changes in perceptions of roles, adherence to norms and subversive challenges to patriarchal structures that often go unaccounted for in the types of M&E instruments often implemented in development projects.

## Concluding points

Social transformation, and in particular gender transformation, is now a central component in resilience‐building. Much of the grey literature on gender‐transformative approaches has explored this topic through community‐level (Promundo‐US and CGIAR Research on Aquatic Agricultural Systems, 2016), institutional (Batliwala and Pittman, 2010; Hillenbrand et al., 2015) and scholarly transformations (Ferdous et al., 2015; McDougall et al., 2015). Cole et al. (2014) explain that transformation is inherently a multi‐scalar process by means of which power structures at varying levels of society are challenged; these include power relationships that exist at the interpersonal and institutional levels, as well as the power embedded in systems of knowledge. Where practitioners can incorporate transformation across each of these areas, there is opportunity for meaningful social change that can enhance resilience. Among scholars and practitioners alike, there is an interest in the creation of spaces and methods to deconstruct and challenge systems of oppression and exclusion, and in turn to facilitate the negotiation and construction of new social norms that allow for the types of agency necessary to respond to the emerging environmental challenges of our time.

Our research contributes to the growing interest in the field of development in identifying mechanisms that lead to transformation at these varying levels of intervention—particularly within multi‐stakeholder partnerships. The two BRACED consortia struggled to find a balance that satisfied a need for flexibility to account for cultural and institutional differences but also a unified strategic plan for achieving transformation. However, at the same time, multi‐stakeholder consortia like BRACED have a unique opportunity to share knowledge and experiences at varying levels of programming and implementation. The knowledge exchange experienced through the BRACED gender workshop explored in this article demonstrates that, through these types of multi‐stakeholder workshops, identification of collective challenges and successes can help synthesise a way forward for development practice.

The analysis of BRACED activities detailed above aims to contribute to a more unified understanding of gender goals and needs at both the organisational and household levels through transformative approaches to resilience‐building. This article describes just one iteration of partner collaboration on gender programming: the nature of the BRACED consortia—with their many moving parts and collaborating partners—made them a challenging space for collective and unified achievement of transformative goals. But the complexity of resilience‐building also means that it offers promising opportunities if donors and programme management facilitate and support spaces for collaboration. Many questions about the potential for gender programming in the two BRACED projects remain. Among the most pressing is whether a gender‐transformative approach is a feasible option for consortia projects like BRACED's over the course of a three‐year timeline. How much transformative change can occur during such a short period? One partner explained that transformation might not be a feasible option for BRACED, as challenging social norms and adopting new practices and belief systems can be a very slow process:

*We have to be very modest when we talk about gender … it is very hard to monitor or quantify. It is about the culture and mentality of people—we can only promote certain ways of thinking. You also need to make sure that you do not disrupt power relations too hard and too fast or it can have a negative backlash effect on these women, it is about planting a seed* (BRACED partner, 2015; Skype interview).

The findings from BRACED suggest that, while our timeframe may be too short to see the outputs of transformative change in gender relations, it may present enough time to begin to lay the foundation for such changes to occur, given the right support system to maintain and improve upon changes in gender equity achieved during the course of the project. The research shows that there are difficulties in operationalising, framing, measuring and evaluating the impact of activities aimed at challenging power inequalities and strengthening resilience. In response, this article highlights the need to have mechanisms and tools in place within M&E and learning processes, to fully capture these nuanced and subtle experiences of nascent change.

Building resilience through reducing social inequality must address the complexity of social relationships, including the vested interests of dominant power structures. Essential to this process is the recognition that pathways to resilience will not be a one‐size‐fits‐all approach, but rather one that is deeply dependent upon context. In fact, strategies for building resilience often vary between members of the same households, across households and, even more importantly, at larger scales. At the same time, these varying strategies are dynamic and shift with the transformation of norms, structures and the associated needs resulting from severe climate events.

In order to be responsive to people's changing needs, multi‐stakeholder consortia must orient their own approaches towards transformation. If the goal is to build resilience, then a gender‐transformative approach is one critical entry point. It is increasingly apparent that addressing gender inequalities has important potential to challenge and transform the underlying vulnerabilities currently hindering resilience efforts. It is not enough to merely treat the expression of those gender inequalities (such as poverty, illiteracy, victimisation through violence) with development interventions; transformation of the underlying causes for that inequality—namely norms and structures of exclusion—is imperative. In transforming the power relationships between men and women, new opportunities in building resilience emerge.

These types of critical transformations can be supported only when development institutions recognise their importance and prioritise processes that allow them to occur. Spaces for multi‐stakeholder relationship‐building and learning, combined with flexibility and adaptive management of time and resources, will lead to better integration of gender‐transformative approaches in building resilience programming. This requires a rethinking of stakeholder relationships towards emphasising opportunities for knowledge‐sharing, social learning and iterative processes of reflection; such processes will be the key to developing flexible and adaptive resilience programming. This review of BRACED activities has shown approaches that fell short of satisfying this need, as well as approaches that did foster such opportunities for intra‐consortia learning, reflection and planning. While initial and ‘on paper’ directives in project planning did not always allow for learning, adaptation and flexibility to occur, the multi‐stakeholder workshops were more successful. Lessons learned from this process should be recognised and considered in future development programmes seeking to support resilience through transformation.

## Acknowledgements

This research emerges from the BRACED Building Resilience and Adaptation to Climate Extremes and Disasters programme funded by DFID and more specifically the Burkina Faso and Ethiopia Christian Aid‐led projects. We would like to thank our partners and all the participants who accepted to share their insights by taking part in interviews as well as the reviewers. The views expressed in this paper are in the authors’ personal capacity, and do not represent the views of their respective institutions. Any errors or omissions remain our own.
